# Two mild phenotype molybdenum cofactor deficiency patients with novel *MOCS2* mutation and immunological treatment after COVID-19 infection

**DOI:** 10.1186/s12883-026-04697-9

**Published:** 2026-02-23

**Authors:** Zhen Shi, Jun Zhu, Binbin Cao, Yang Tian, Jie Yu, Chi Hou, Haixia Zhu, Xiuying Wang, Bingwei Peng, Yani Zhang, Kelu Zheng, Xiaojing Li, Yuanyuan Gao

**Affiliations:** 1 Department of Neurology, Guangzhou Women and Children’s Medical Center, Guangzhou Medical University, 9# Jin Sui Road, Guangzhou, 510623 China; 2 South China Advanced Institute for Soft Matter Science and Technology (AISMST), School of Emergent Soft Matter, South China University of Technology, Guangzhou, 510640 China; 3 Department of Neurology, Guangzhou Women and Children’s Medical Center Liuzhou Hospital, Liuzhou, 545000 China

**Keywords:** Molybdenum cofactor deficiency, Mild phenotype, Molybdenum cofactor synthesis gene 2, COVID-19, Cytokine, Immunological therapy

## Abstract

**Background:**

Molybdenum cofactor deficiency type B (MoCD-B) is a rare autosomal recessive metabolic disorder caused by mutations in *MOCS2*. Patients with mild phenotypes may experience infection-related neurological deterioration followed by partial spontaneous recovery. This study presents the first reported cases of acute encephalopathy triggered by SARS-CoV-2 infection in patients with mild MoCD-B and provides clinical insights into the course of neurological symptoms and corresponding interventions.

**Methods:**

Clinical and genetic information were collected from two patients showing acute encephalopathy, each harboring mutated molybdenum cofactor synthesis gene 2 (*MOCS2*).

**Results:**

Both patients developed acute encephalopathy following SARS-CoV-2 infection. Clinical manifestations included motor regression, dystonia, and brain MRI abnormalities predominantly involving the bilateral globus pallidus and cerebral peduncles. Biochemical testing revealed persistently low serum uric acid and hypohomocysteinemia. Immunological analysis showed positive thyroid autoantibodies. Cerebrospinal fluid (CSF) analysis indicated elevated levels of interleukin-8 and other pro-inflammatory cytokines during the acute phase. Whole-exome sequencing identified a known pathogenic variant (c.16 C > T, p.Gln6Ter) and a novel missense variant (c.257G > T, p.Ser86Ile) in *MOCS2*. Both patients received immunotherapy with intravenous immunoglobulin (IVIG) and methylprednisolone, resulting in gradual symptom improvement.

**Conclusion:**

This study expands the genetic spectrum of MoCD-B by identifying for the first time a novel *MOCS2* variant (c.257G > T, p.Ser86Ile). In infants, acute neurological deterioration after infection accompanied by persistent hypouricemia and bilateral globus pallidus lesions should prompt consideration of mild MoCD-B. Our observations do not provide sufficient evidence to support routine immunotherapy for MoCD-B patients after SARS-CoV-2 infection; treatment management should be guided by established recommendations for COVID-19–associated neurological complications.

**Clinical trial number:**

Not applicable.

**Supplementary Information:**

The online version contains supplementary material available at 10.1186/s12883-026-04697-9.

## Introduction

 Severe acute respiratory syndrome coronavirus 2 (SARS-CoV-2), which emerged in 2019, has been widely acknowledged for its ability to induce multisystem inflammation and tissue injury. In addition to pulmonary diseases, neurological complications consist of one of the major causes of hospitalization in the pediatric population following SARS-CoV-2 infection. In individuals carrying specific genetic mutations, SARS-CoV-2 infection appears to predispose to neurological manifestations. For example, patients with Ran Binding Protein 2 (*RANBP2*) mutations are more susceptible to developing acute necrotizing encephalopathy (ANE) following viral infections such as influenza and COVID-19, with an extremely high serum interleukin-6 (IL-6) level [1]. Together, these observations indicate that intercurrent infections can trigger neuroinflammatory responses and precipitate neurological deterioration in genetically predisposed individuals, providing a clinical perspective for considering infection-associated worsening in other neurological disorders, including molybdenum cofactor deficiency (MoCD).

MoCD is a rare autosomal recessive disorder, which can be classified into two forms. The typical, early-onset type manifests within 24–72 h after birth with severe neurological symptoms, including intractable seizures and a classical electroencephalogram (EEG) pattern-burst suppression. Brain magnetic resonance imaging (MRI) commonly demonstrates encephaledema involving cerebral cortex, basal ganglia and white matter [2], some with progressive atrophy. The atypical, or mild phenotype, presents later in infancy with developmental delay or regression, often triggered by intercurrent infections. Development can be normal or slightly delayed, but regression and fluctuating dystonia are often triggered by infectious diseases. Some patients can achieve motor and language capability, though expressive language difficulties are common. Brain MRI showed bilateral abnormalities in the globus pallidus and cerebellar dentate nucleus [3, 4]. Biochemical tests typically revealed hypouricemia and elevated urinary sulfite levels [4–6]. MoCD is caused by mutations in four genes including molybdenum cofactor synthesis gene 1 (*MOCS1*), molybdenum cofactor synthesis gene 2 (*MOCS2*)/molybdenum cofactor synthesis gene 3 (*MOCS3*) and gephyrin gene (*GEPH)*, which involved in molybdenum cofactor (Moco) biosynthesis, related to MoCD type A, type B and type C, respectively [7]. Despite genetic heterogeneity of MoCD, clinical manifestations are largely indistinguishable among the three types. *MOCS1* mutations account for approximately 50% of cases, followed by *MOCS2*, which is responsible for 45%. *MOCS2* encodes two transcripts, *MOCS2A* (molybdenum cofactor synthesis gene 2A) and *MOCS2B* (molybdenum cofactor synthesis gene 2B), which together form molybdopterin (MPT) synthase, an essential enzyme converting cyclic pyranopterin monophosphate (cPMP) to MPT [7, 8]. Dysfunction of MPT synthase due to *MOCS2* mutations leads to a failure in Moco biosynthesis, impairing function of Moco-dependent enzymes such as xanthine dehydrogenase, aldehyde oxidase [2], sulfite oxidase, mitochondrial amidoxime reducing complex (mARC) [9, 10], leading to two pathogenic mechanisms-impaired purine degradation caused by disfunction of xanthine dehydrogenase/aldehyde oxidase, and disrupted sulfur detoxification due to disfunction of sulfite oxidase. The former is typically associated with characteristic purine abnormalities and recurrent urolithiasis but is generally not associated with neurological manifestations, whereas the latter leads to the accumulation of toxic metabolites, such as sulfite and S-Sulfocysteine (SSC) [10, 11], resulting in progressive neurological damage. In patients with mild MoCD, disease onset or neurological deterioration is frequently precipitated by intercurrent infections. However, the mechanisms by which infection acts as a trigger for MoCD remain poorly understood.

In this study, we report two patients with MoCD-B harboring biallelic *MOCS2* (OMIM #603708) variants who developed acute encephalopathy triggers with SARS-CoV-2 infection. We also conducted a PubMed-based literature review of reported mild MoCD-B cases published between 1990 and June 2025 (Table S1) to summarize clinical and genetic features and to discuss potential mechanisms underlying infection-triggered decompensation in this rare disorder.

## Methods

### Patients

This retrospective case-series study was conducted at Guangzhou Women and Children’s Medical Center. The hospital’s electronic medical record system was searched from January 2018 to July 2024 to identify patients with suspected MoCD. Inclusion criteria were: Clinical manifestations compatible with MoCD, including acute or recurrent encephalopathy after infection, seizures, dystonia or choreoathetosis, fluctuating muscle tone, or developmental delay/regression. Biallelic *MOCS2* variants identified by whole-exome sequencing (WES) and classified as variants of uncertain significance (VUS), pathogenic or likely pathogenic according to ACMG guidelines. Exclusion criteria was an alternative diagnosis that fully explained the neurological phenotype.

### Clinical data collection

Demographic and clinical information was collected, including: Age at onset and triggering factors, seizure, movement abnormalities, muscle tone fluctuations, pyramidal signs, and developmental milestone, family history and perinatal history.

clinical course after SARS-CoV-2 infection, response to immunotherapy (IVIG and/or methylprednisolone) and follow-up visiting. The following laboratory measurements were collected, including serum uric acid, plasma amino acids, plasma total homocysteine, urinary organic acid analysis, X-ray imaging, MRI, EEG, as well as additional examinations as clinically indicated. Analyses were performed using standard automated biochemical analyzers in the hospital central laboratory. The information of all mild patients with MoCD-B recorded in Pubmed was reviewed [3, 4, 12–20].

### Cytokine analysis

Cerebrospinal fluid (CSF) was collected through lumbar puncture into sterile polypropylene tubes. Samples were centrifuged at 400 ×g for 10 min at 4 °C to pellet cells and debris. The supernatant was carefully aliquoted into low-protein-binding microtubes and stored at − 4 °C. A commercial bead-based multiplex cytokine detection kit (Aimplex, QuantoBio, Beijing, China) was used for simultaneous quantification of 14 cytokines: IFN‑γ, IL‑1β, IL‑2, IL‑4, IL‑5, IL‑6, IL‑8, IL‑10, IL‑12p70, IL‑17 A, IL‑17 F, IL‑22, TNF‑α, and TNF‑β. The kit included capture antibody-coated fluorescent microspheres, biotinylated detection antibodies, and PE-conjugated streptavidin (SA‑PE). All assays were performed on a BD FACSCanto II flow cytometer equipped with a 488 nm laser for PE detection. Samples were collected at 19 and 23 days after infection onset (Table 1). The reference values for cytokines were supplied by the manufacturer and were documented in Supplementary Table S2. The detailed procedure adhered to the protocol established has been published [21].

**Table 1:** Results of biochemical markers detected in two patients in this study

Patient ID	Plasma uric acid (90–460 umol/L)	Homocysteine^#^ (umol/L)	Serum amino acid (umol/L)			Cytokine in the CSF (pg/ml)		TBNK lymphocytes subsets		Anti-thyroid antibodies (IU/ml)	
				(D 11)	(D 19)	(D 19)				(D 15)	
Pt 1	<21	<3	homocysteine	0.73	1.06	TNF-β	3.81			TGA 154.61	
			(4.4–10.6)			(0-2.54)				TPO 58.77	
			cysteine	42.19	53.46	TNFα	4.72	/			
			(123.3–213)			(0-4.5)					
			glutathione (whole) (4.3–12.6)	1.34	2.39	IL-8	50.19				
						(0-15.71)					
			glutamine	623.54	569.72						
			(287.7–588)								
				(D 11)		(Day 23)				(D 7)	(D 34)
Pt 2	<21	<3	aspartic acid (18–47)	50.5		IL-1β	4.9	T cell ratio (50–84%)	49.16	TPO<5	TPO 42.11
						(0-3.4)				TGA<10	TGA 54.03
			asparagine (1–12)	25.6		IL-4	7.82	B cell ratio (5–18%)	32.54		
						(0-4.19)					
			glutamic acid (24–189)	361.1		IL-5	5.96	NK cell ratio (7–40%)	15.79		
						(0-4.15)					
						IL-6	12.01	CD8 + T cell (190–1140) cell/ul	217.27		
						(0-11.09)					
						IL-10	6.72	CD4 + T cell (410–1590) cell/ul	297.14		
						(0-4.5)					
						IL-17 A	4.83	CD8 + Tcell ratio (13–41%)	11.43		
						(0-4.74)					
						IL-22	4.31	CD4+/CD8 + ratio (0.68–2.47)	31.42		
						(0-3.64)					
						TNFα	7.7				
						(0-4.5)					
						IL-8	52.12				
						(0-15.71)					
						IFN-γ	10.6				
						(0-4.43)					

^#^ Tested by enzyme cycling assay method

*Abbreviations CSF* cerebrospinal fluid, *ATG* antithyroid glubulin antibody, *TPO* antithyroid peroxidase antibody

### Consents

Informed consents were obtained from the parents of the children. This study was approved by the Human Research Ethics Committee of Guangzhou Women and Children’s Medical Center.

### Genetic analysis

Peripheral venous blood was collected from two probands and their parents using EDTA anticoagulant tubes. Genomic DNA was extracted using the RelaxGene Blood DNA System (Tiangen Biotech Co., Ltd., Beijing, China). Sequencing libraries were prepared with the NanoPrep DNA Library Preparation Kit (for MGI, 96 reactions) and sequenced on a BGI MGISEQ-2000 instrument to generate 2 × 150 bp paired-end reads, achieving a minimum coverage depth of 150×. Raw reads were aligned to the human reference genome (hg38/GRCh38) using the Burrows-Wheeler Aligner (BWA v0.7.17) with default parameters [22]. The resulting BAM files were sorted and deduplicated using SAMtools [23] and Picard, respectively. Single nucleotide variants (SNVs) and short insertions/deletions (Indels < 50 bp) were called using the Genome Analysis Toolkit (GATK) [24]. Identified variants were annotated for population frequency using the 1000 Genomes Project, Genome Aggregation Database (gnomAD), and Exome Aggregation Consortium (ExAC). Disease associations were annotated based on the Online Mendelian Inheritance in Man (OMIM), Human Gene Mutation Database (HGMD), and ClinVar databases. The functional impact of variants was predicted using multiple tools: PolyPhen-2, SIFT, MutationTaster. Candidate pathogenic variants were finally prioritized according to the guidelines of the American College of Medical Genetics and Genomics (ACMG). PCR-Sanger sequencing was performed to validate the candidate pathogenic variants.

### Crystal structures analysis

The original human molybdopterin synthase complex (PDB ID: 5MPO), including MOCS2A (PDB ID: O96033) and MOCS2B (PDB ID: O96007), was obtained from UniProt. The mutated MOCS2A structures were predicted using AlphaFold2 (https://neurosnap.ai) and analyzed with PyMOL 3.0.5 software and ChimeraX. Missense variants in *MOCS2A* (affecting the MOCS2A subunit) reported in patients with MoCD-B were mapped onto the MOCS2A–MOCS2B complex using an AlphaFold2-derived model. Structural consequences were analyzed using both ChimeraX for superimposition and visual comparison of wild-type and mutant models, and PyMOL for detailed examination of local interactions, such as hydrogen bonds.

## Results

### Patient data

Patient 1 was the second child of a non-consanguineous Chinese couple in southern China. She was born at term, through cesarean delivery due to macrosomia, with a birth weight of 4.35 kg. Her mother had gestational diabetes mellitus. The patient achieved stable head control at the age of 3 months, and turned over aged 4 months, but failed to sit independently at 6 months of age.

**Fig. 1:**
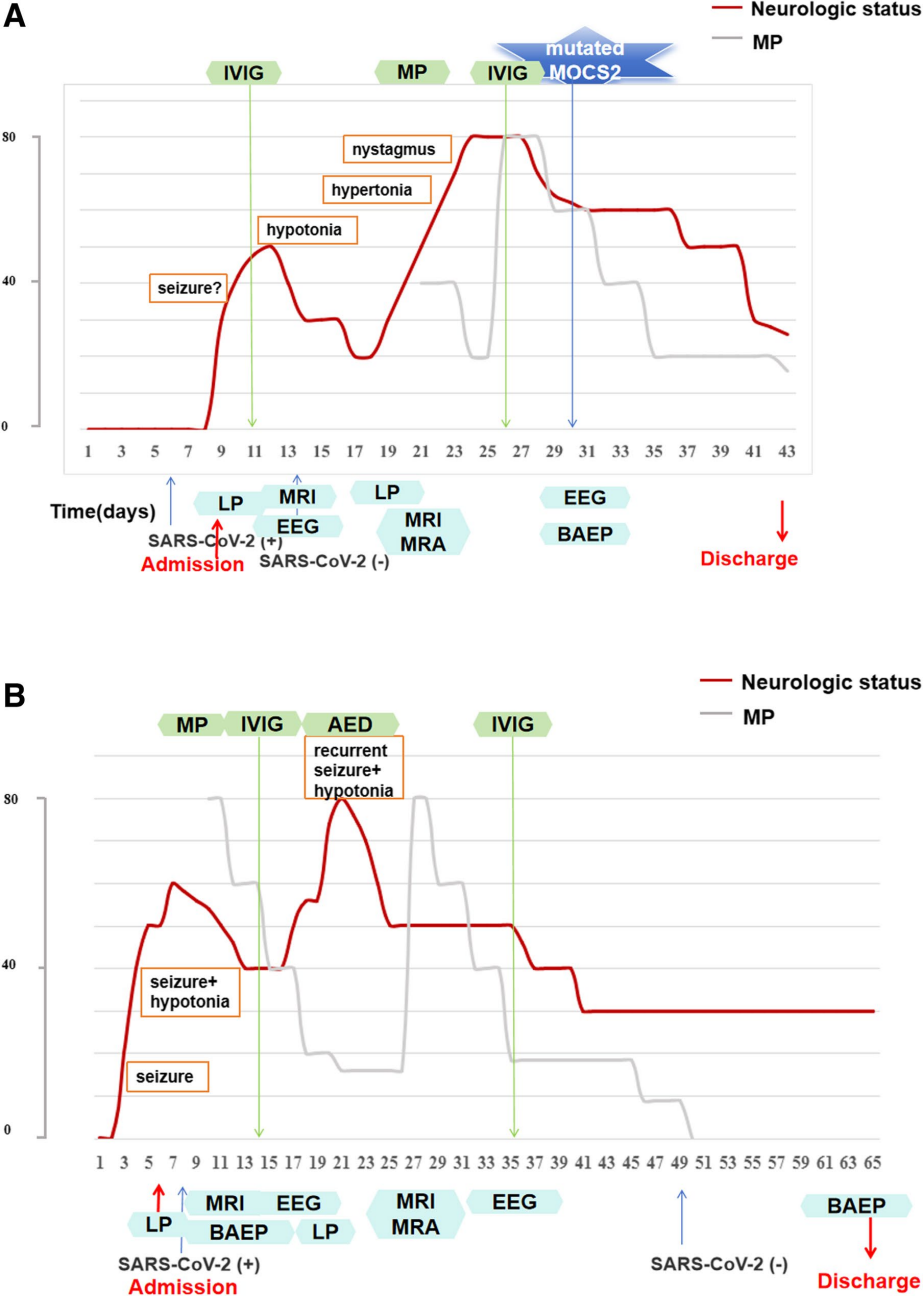
Clinical course of two patients with mild MoCD-B following SARS-CoV-2. (**A**) Clinical course of Patient 1. (**B**) Clinical course of Patient 2

**Fig. 2:**
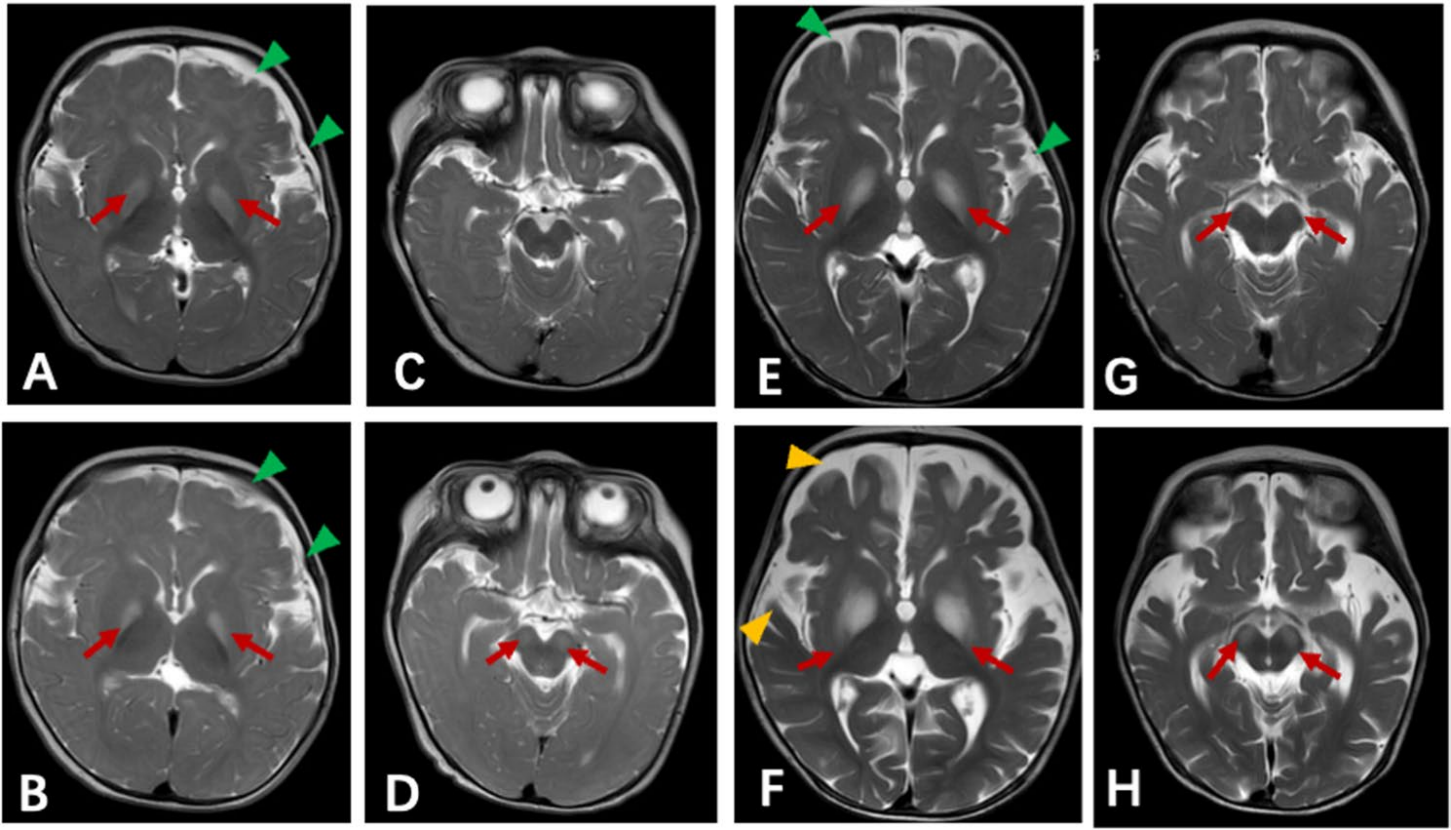
Brain MRI findings in two patients with mild *MOCS2*-related MoCD-B. Axial T2-weighted images of Patient 1 (**A**–**D**) and Patient 2 (**E**–**H**). Top panels (**A**, **C**, **E**, **G**) were obtained at disease onset, and bottom panels (**B**, **D**, **F**, **H**) on repeat MRI during the same hospitalization. Red arrows indicate symmetric hyperintensities in the bilateral globi pallidi and cerebral peduncles. Green and yellow arrowheads indicate enlarged extracerebral spaces

At the beginning (Day 1 – Day 7), the patient had visited our out clinic and emergency room for several times, due to persistent high fever and irritability, and the upper respiratory virus nucleic acid testing revealed she was infected with COVID-19. Unpredictably (Day 8), she developed motor regression, abnormal posture, and suspicious seizures (Fig. 1A, Table S1), thus she was admitted to the infection ward in our hospital (Day 9). Physical examination showed reduced movement in right limbs and hypertonia, hyperreflexia and positive ankle clonus. Initial laboratory tests, including blood infectious markers, cytologic and biochemical results of cerebrospinal fluid (CSF) analysis were unremarkable (Day 9-Day 10). Meanwhile, the 1st MRI (Fig. 2, S2) demonstrated swollen bilateral globus pallidus with hyperintense signals on T2-weighted imaging and restricted diffusion on diffusion weighted imaging (DWI) and apparent diffusion coefficient (ADC). Therefore, COVID-19 associated encephalopathy was suspected, and intravenous immunoglobulin (IVIG; 2 g/kg, Day 11) was administered. To differentiate metabolic and vasogenic disease, serum amino acid and urinary organic acid analysis, as well as magnetic resonance angiography (MRA) were conducted. On Day 12, hypotonia and reduced deep tendon reflexes developed. Although clinical symptoms initially improved, a second MRI on Day 11 showed reduced swelling in the bilateral globus pallidus but new hyperintense signals in bilateral cerebral peduncles. MRA ruled out vasogenic abnormalities. Brainstem auditory evoked potential (BAEP) indicated mild right-sided injury. A re-evaluation of CSF analysis was performed (autoimmune encephalitis antibodies and oligoclonal bands were negative), and cytokine analysis in the CSF (Day 19) demonstrated elevated IL-6 and interleukin-8 (IL-8) (Table 1), indicating inflammatory activation. Thus, a small dosage (40 mg/d, 5 mg/kg/d) of methylprednisolone (MP) was administered.

On the second day after the treatment (Day 22), she exhibited irritability, feeding difficulty, vertical nystagmus, hypertonia, and hyperreflexia in upper limbs, followed by loss of visual tracking, smiling, or responding to sound, consistent with the new MRI changes. MP was reduced to 2.5 mg/kg/d after using 3 days due to suspicious side effect-irritability. Nasogastric tube feeding was initiated, and nitrazepam (0.176 mg/kg.d) was added to alleviate hypertonia.

Serum full spectrum amino acids analysis revealed decreased cysteine, homocysteine, glutathione, ornithine, tryptophan, histidine, lysine and phenylalanine, and elevated glutamine. Given suspected hereditary factor, trio whole-exome sequencing (WES) was performed with parental consent.

Concurrently, anti-thyroid antibodies (TGA: 154.6 IU/ml; TPO: 58.77 IU/ml) were elevated, and EEG revealed slow background wave pattern with intermittent right temporal spikes. Although the patient’s condition fluctuated, the first course immunological treatment was effective. Therefore, a second course immunological treatment, consisting of 2 g/kg IVIG (400 mg/d, 5 days) and high dose of MP (10 mg/kg.d), was initiated on Day 26. Serum and CSF lactate levels were slightly elevated. While the serum lactate/pyruvate ratio remained within the normal range, the ratio in the CSF was mildly increased. Therefore, supplementary therapy with an energy mixture (coenzyme Q10, thiamine, riboflavin, pyridoxine) was introduced to support mitochondrial function.

On Day 33, visual and auditory tracking improved. Her sucking ability returned, with slight nystagmus, mild lower limb hypotonia and upper limb hypertonia, concurrently, prednisone reduced gradually. Despite these improvements, EEG abnormalities and BAEP injury persisted.

On Day 35, trios WES identified a homozygous *MOCS2* variant c.16 C > T(p.Gln6Ter), inherited from her healthy parents. Mitochondrial DNA (mtDNA) sequencing was negative. Serum uric acid was extremely low (Table 1). The patient stabilized with immunological treatments and supportive care. Given the stabilization of clinical symptoms and the lack of disease-specific therapy for MoCD-B, the family decided to discharge (Day 43). At age of 11 months, she could vocalize, with unstable head control, but remained unable to turn over, and showed mild dysphagia.

Patient 2 was the second child of a non-consanguineous Chinese family in southern China, born at term through cesarean delivery due to a scarred uterus. At 3 months, she showed developmental delay and suspected seizures following infection. At 5 months, she first suffered a febrile episode (Day 1), and was admitted to local hospital with weakness and suspected seizures (Day 2- Day 5) (Fig. 1B). Initial cranial MRI was unremarkable, and sedative treatment alleviated her seizures. For further treatment, the patient was transferred to ICU ward in our hospital (Day 6). Her physical examination revealed microcephaly, reduced muscle tone and hyporeflexia. Spinal muscular atrophy and spinal cord disease were ruled out through survival motor neuron gene testing and spine cord MRI initially. Blood tests were normal, and chest X-ray confirmed bronchopneumonia. SARS-CoV-2 PCR was positive (Day 8). On Day 10, MRI displayed hyperintensities in the bilateral globus pallidus and cerebral peduncles on T2-weighted imaging (Fig. 2), BAEP revealed bilateral injury, while EEG was normal. CSF tests showed normal cytology and biochemistry, with negative viral nucleic acid results. Given these findings, COVID-19 associated encephalopathy and inherited metabolic diseases were suspected. The patient was treated with high-flow oxygen support, methylprednisolone (MP; 10 mg/kg/day, Day 10), and IVIG (2 g/kg, D14). She was transferred to neurology ward on Day 14 when the condition became stable.

On Day 16, she presented with frequent limb rigidity and tremor, with EEG showing slow-wave activity. Nitrazepam (0.07 mg/kg/d) and Levetiracetam were added but failed to fully control the seizures even with diazepam or midazolam. Respiratory distress led to re-intubation. The re-examination of brain MRI demonstrated enlarged abnormal signals in swollen bilateral globus pallidus as well as bilateral cerebral peduncles (Fig. 2). Her EEG demonstrated a diffuse background slowing and intermittent slow-wave activity in frontal regions. Repeated BAEP also indicated a worsening condition. A lumbar puncture was re-performed (Day 23), revealing normal cytologic and biochemical results in CSF analysis. However, IL-8 level in the CSF was elevated (50.19 pg/ml), and TPO was mildly increased (Table 1). A second course of MP and IVIG were administered. she developed pneumonia with elevated C-reactive protein, and sputum culture identified *Klebsiella pneumoniae*,

for which antibiotics were administered. She was gradually weaned from the ventilator, and her COVID-19 test remained positive until Day 49. She transferred back to neurology. On Day 46, levetiracetam (35 mg/kg/day) and tapering doses of MP. She could respond to sound and tracking objects, but still required nasogastric feeding and displayed hypotonia, hyporeflexia, stiff facial expressions and chewing unconsciously. Lactic acid was normal, except for transient elevations during respiratory distress. The serum lactate/pyruvate ratio was normal, whereas the CSF ratio was slightly increased, despite normal CSF lactate levels. Persistently low serum uric acid levels, elevated homocysteine in serum amino acids and normal urinary GCMS analysis raised suspicion of MoCD. The family guard finally agreed to perform trios WES and mtDNA testing. However, intermittent rapid breathing occurred, and triple-nebulization with budesonide, salbutamol, and ipratropium bromide provided no improvement. Symptoms of upper airway obstruction appeared, but both the laryngeal airway and chest X-ray were normal. Subglottic stenosis (2 mm) was confirmed by laryngoscopy. Before scheduled surgery, she experienced sudden respiratory depression requiring ICU readmission and mechanical ventilation. Despite stabilization, the family opted to discontinue treatment, and the patient was discharged against the selective operation by otolaryngologist from ICU ward, after counseling regarding prognosis and potential risks. The patient passed away a few days after discharge.

The WES revealed the patient harbored a compound heterozygous mutation in *MOCS2* (c.16 C > T [p.Gln6Ter] and c.257G > T [p.Ser86Ile]), inherited from her healthy father and mother, respectively. Her mitochondrial DNA sequencing was negative.

### Clinical summary

We reviewed 11 patients with mild MoCD-B phenotype. A detailed clinical summary reported in the articles found by the PubMed search is provided in Supplementary Table S1, including demographic data, onset age, clinical manifestations, biochemical findings, neuroimaging features, genotype, treatment, and outcomes.

For Patient 1, at the 11-month follow-up visit, she could take 120–150 mL milk per feed. Excessive oral secretions and drooling were noted. She demonstrated visual and auditory tracking but had poor head control and was unable to roll over, bring her hands to her mouth, or voluntarily grasp objects. She could smile and vocalize but did not distinguish familiar from unfamiliar people.

Neurological function changes around SARS-CoV-2–associated exacerbation in mild MoCD-B in two patients are summarized in Table S3.

### Genetic results

Variants in *MOCS2* were identified in two unrelated patients with mild MoCD-B. Patient 1 carried a homozygous nonsense variant c.16 C > T (p.Gln6Ter), whereas Patient 2 harbored compound heterozygous variants c.16 C > T and c.257G > T (p.Ser86Ile) (Figure S1 and Table 2). The c.16 C > T variant has previously been reported in Chinese patients with MoCD-B, while c.257G > T represents a novel missense variant that has not been described in the literature or in population databases. According to ACMG guidelines for variant interpretation, c.16 C > T is classified as pathogenic, and c.257G > T is classified as likely pathogenic (PM2 + PM3 + PP3 + PP4).

**Table 2:** Missense *MOCS2* variants with pathogenicity analysis

	Nucleotide alteration	Location	Amino acid alteration	gnomAD-EA	Han Chinese genomes	Mutation Taster	SIFT	PolyPhen-2	PROVEAN	FATHMM	GERP
MOCS2A	c.257G > T	5:52402935	*p*.Ser86Ile	-	-	Disease causing (0.5228)	D (0)	Probably damaging (0.999)	Damaging (0.87611)	Damaging (0.99035)	Conserved (6.02)
	c.19G > T	5:53108643	*p*.Val7Phe	5.05E-05	9.925E-5	Disease causing (0.9995)	D (0)	Possibly damaging (0.194)	Neutral (0.52612)	Damaging (0.93858)	Conserved (4.02)
	c.45T > A	5:53108617	*p*.Ser15Arg	-	1.2127E-4	Disease causing (0.9737)	D (0.02)	Probably damaging (0.968)	Damaging (0.57275)	Damaging (0.82593)	Non-conserved (-0.508)
	c.218T > C	5:53107144	*p*.Leu73Pro	-	-	Disease causing (1)	D (0)	Probably damaging (0.931)	Damaging (0.75935)	Damaging (0.99192)	Conserved (5.75)
	c.226G > A	5:53107136	*p*.Gly76Arg	-	-	Disease causing (1)	D (0.03)	Possibly damaging (0.717)	Damaging (0.79316)	Damaging (0.98428)	Conserved (4.88)
	c.244 A > T	5:53107118	*p*.Ile82Phe	1.315E-5	-	Disease causing (0.6857)	D (0.02)	Benign (0.012)	Damaging (0.66206)	Damaging (0.98932)	Conserved (5.88)
	c.263G > C	5:53107099	p.Gly88Ala	-	-	Polymorphism (0.648)	D (0.02)	probably damaging (0.999)	Damaging (0.8794)	Damaging (0.75)	Conserved (6.02)
*MOCS2B*	c.564G > C	5:53101359	p.Gly126Ala	-	-	Disease causing 1	D (0.01)	Probably damaging (1)	Damaging (0.87063)	Damaging (0.9832)	Conserved (5.83)

*Abbreviations*, principles and cutoff values of in silico prediction tools: *EA* East Asian, *D* Deleterious, *FATHMM* Functional Analysis through Hidden Markov Models Uses hidden Markov models and evolutionary conservation to predict functional impact

Cutoff values: Damaging ( ≤ − 1.5), Tolerated ( > − 1.5); GERP (Genomic Evolutionary Rate Profiling): Measures evolutionary constraint by comparing observed vs. expected substitution rates. Cutoff values: Highly conserved (> 4), Moderately conserved (2–4), Neutral (0–2), Fast-evolving (< 0); MutationTaster: Bayesian classifier predicting disease-causing potential. Categories include: disease causing, disease causing automatic (known pathogenic), polymorphism, and polymorphism automatic (known benign). Provides a probability score; values close to 1 indicate high confidence; NA, not available; PolyPhen-2 (Polymorphism Phenotyping v2): Predicts functional impact based on evolutionary conservation, protein 3D structure, and functional annotations. Cutoff values: Probably damaging (0.85–1.00), Possibly damaging (0.15–0.85), Benign (0.00–0.15); PROVEAN (Protein Variation Effect Analyzer): Predicts whether amino acid substitutions or indels affect protein function based on sequence similarity scores. Cutoff values: Deleterious ( ≤ − 2.5), Neutral ( > − 2.5); SIFT (Sorting Intolerant From Tolerant): Evaluates conservation of amino acids across species; highly conserved sites are intolerant to substitutions. Cutoff values: Deleterious (≤ 0.05), Tolerated (> 0.05)

We reviewed all *MOCS2* variants reported in patients with mild MoCD-B. To date, seven variants have been detected in this phenotype (Table S1), including c.3G > A (p.Met1Ile), c.16 C > T (p.Gln6Ter), c.19G > T (p.Val7Phe), c.257G > T (p.Ser86Ile), c.263G > C (p.Gly88Ala), c.564G > C (p.Gly126Ala), and c.726_727delAA. Among these, c.16 C > T (p.Gln6Ter), c.3G > A (p.Met1Ile) and c.19G > T (p.Val7Phe) were the most frequent, accounting for 30.7%, 23.1% and 19.2% of the total variant allele count, respectively. Based on published variants and our own cases, seven missense variants in *MOCS2A* and one missense variant in *MOCS2B* (c.564G > C) have been described in patients with MoCD-B. Because both variants identified in our study are predicted to affect the MOCS2A subunit, we restricted our structural analyses to missense variants impacting *MOCS2A* and did not model variants affecting *MOCS2B*. Seven *MOCS2A* missense variants include three associated with mild phenotypes (p.Ser86Ile, p.Val7Phe, p.Gly88Ala) and four severe phenotypes (p.Leu73Pro, p.Gly76Arg, p.Ile82Phe, p.Ser15Arg). Mutated variants were analyzed using the online Variant Effect Predictor (VEP) (Table 2).

### Structural analysis

**Fig. 3:**
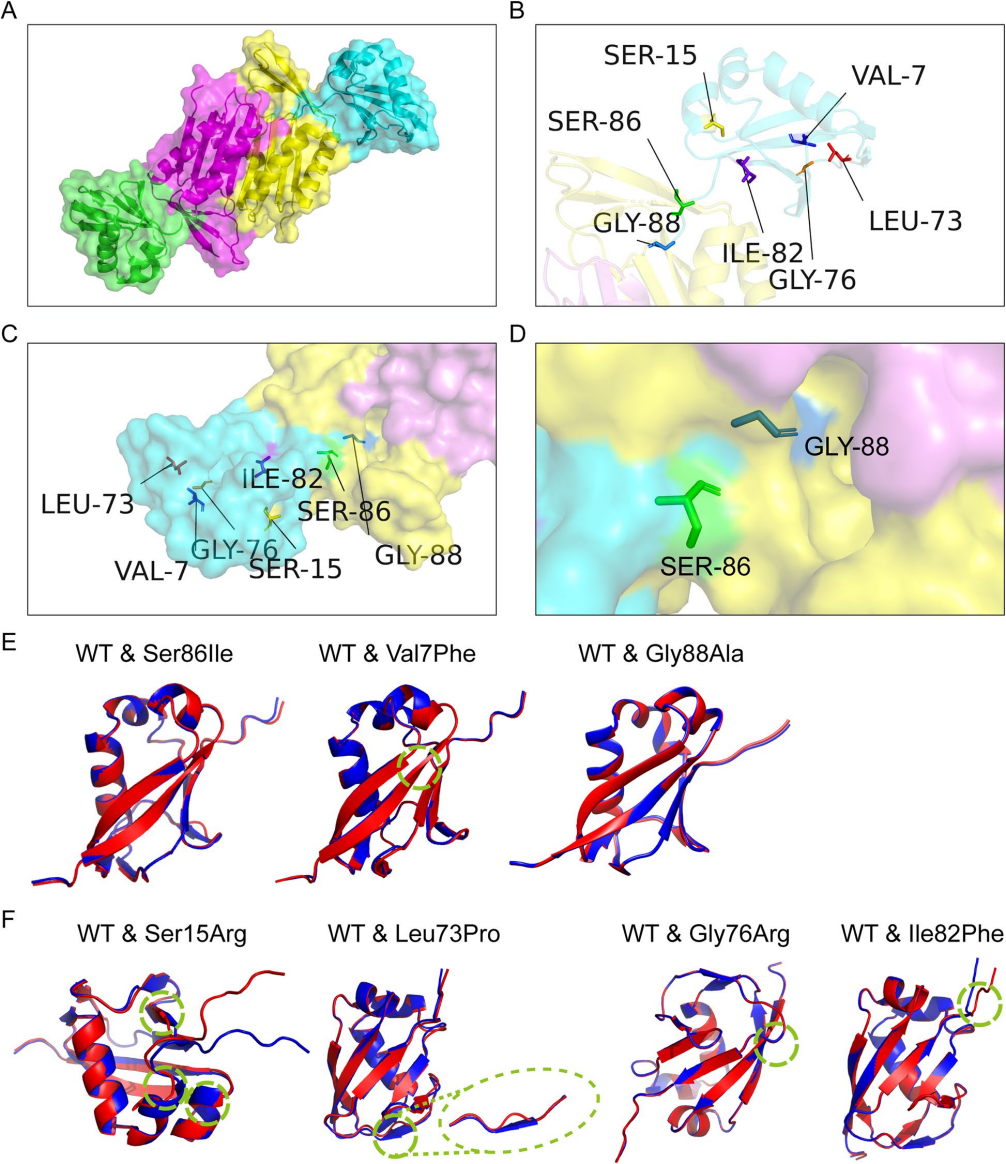
Localization of MOCS2A missense variants on the MOCS2A–MOCS2B complex and comparison of MOCS2A-WT protein structure with its mutant forms. (**A**) Schematic illustration of theMPT synase (MOCS2A–MOCS2B heterotetramer). (**B**) Locations of the seven missense variants mapped onto the MOCS2A structure (cartoon representation). (**C**) The MOCS2A–MOCS2B interface shown in a surface representation of the complex. (**D**) Close-up view highlighting Ser86 and Gly88 residues located at the MOCS2A–MOCS2B interface. **E**. Superimposition of the WT protein (red) and its three variants associated with mild phenotype (blue): p.Val7Phe, p. Ser86Ile and p. Gly88Ala. (**F**) Superimposition of the WT protein (red) and its four variants associated with severe phenotype (blue): p.Leu73Pro, p.Ile82Phe, p.Gly76Arg, and p.Ser15Arg. Structural changes induced by each mutation are highlighted within green dashed circles, showing local conformational differences between the WT and mutant forms

To explore the potential protein structural effect of missense variants, we first performed a literature review to collect all reported missense variants in *MOCS2*. In addition to the novel missense variant c.257G > T (p.Ser86Ile) identified in this study, we identified seven previously reported missense variants, including six in *MOCS2A* and one in *MOCS2B* (c.564G > C). Because both variants identified in our study are predicted to affect the MOCS2A subunit, we focused our structural analyses on the *MOCS2A* missense variants. We generated an AlphaFold2-based model of MOCS2A protein. Variant locations and predicted secondary-structure features of MOCS2A variants were examined in ChimeraX (Fig. 3; Table 3), with structural intermolecular measurements performed in PyMOL (Fig. S3; Table 3). Two MOCS2A and two MOCS2B subunits assemble into a functional heterotetrameric MPT synthase in PyMOL (Fig. 3A). The p.Ser86Ile and p.Gly88Ala mutations are located on the interface of MOCS2A and MOCS2B (Fig. 3C and D), proximal to or within the C terminal Gly-Gly motif (active sites). In ChimeraX analysis, the p.Ser86Ile, p.Gly88Ala and p.Val7Phe mutation showed minor impact on the overall protein structure(Fig. 3E), whearas p.Leu73Pro, p.Gly76Arg, p.Ile82Phe and p.Ser15Arg demonstrated significant impacts on the crystal structure, whose C-terminal region deviates from that of wild-type (WT) MOCS2A (Fig. 3F). In our PyMOL-based analysis (Table 3, Fig. S3), the number of hydrogen bonds formed by Val7, Gly76, and Ile82 with neighboring residues remained unchanged between the wild-type and variant models. In contrast, the number of hydrogen bonds decreased after substitution of Ser15 with arginine and Leu73 with proline. For p.Ser86Ile and p.Gly88Ala, no hydrogen bonds were formed between the mutated residues and other MOCS2A residues in our structural model (data didn’t show).

**Table 3:** Predicted structural alterations of missense MOCS2A variants identified in mild MoCD-B

Variants of MOCS2A (HGVS protein)	Location;	Located distributaion away to active sites	Located on interface between MOCS2A-MOCS2B	Number of hydrogen bonds (pre/post mutation)	Structural superposition (ChimeraX)	Literature structural prediction evidence	Literature evidence (biochemical experiments performed? )	Clinical phenotype
p.Ser15Arg	N-terminal	Distant from active sites	No	↓ (7 − 5)	Largest local displacement	Not reported	Minimal amounts of MOCS2A-S15R could be obtained after purification. (Leimkühler et al., 2003)	severe
p.Leu73Pro	C-terminal	Near active sites	No	↓ (1 − 0)	Displacement	Not reported	Not reported	severe
p.Gly76Arg	C-terminal	near C terminal active tail	No	Unchanged (1)	Displacement	Not reported	Not reported	severe
p.Ile82Phe	C-terminal	Proximal to C terminal active tail	No	Unchanged (1)	Displacement	Not reported	Not reported	severe
p.Val7Phe	N-terminal	Distant from C terminal active tail	Unchanged	Unchanged (2)	Slight displacement	Not reported	Yes. (1) SEC indicates V7F fails to form a complex with MOCS2B. (2) In vitro activity ~ 10% and formation of a half-sulfurated intermediate. (3) CD Spectroscopy differences vs. WT. (Leimkühler et al., 2003)	mild
p.Ser86Ile	C-terminal	Within C terminal active tail	yes/promixal to terminal Gly-Gly motif	None	Slight displacement	Not reported	Not reported	mild
p.Gly88Ala	C-terminal	Within C terminal active tail	yes/the last terminal Gly-Gly motif	None	Slight displacement	Not reported	Not reported	mild

*Abbreviations Ala* alanine, *AMP* adenosine monophosphate, *CD* Circular Dichroism, *Gly* Glycine, *SEC* Size-exclusion chromatography

Location and secondary-structure changes were annotated on the AlphaFold2 MOCS2A model in ChimeraX, and all distances were measured in PyMOL

## Discussion

*MOCS2* encodes two protein subunits: a small subunit (MOCS2A, 88 amino acids) and a large subunit (MOCS2B, 188 amino acids), respectively. These two subunits assemble into a functional A_2_B_2_ heterotetrameric MPT synthase [25]. In this complex, two MOCS2A subunits act as sulfur carriers via their C-terminal thiocarboxylated Gly–Gly motifs [26], whereas two MOCS2B subunits form the catalytic pocket that converts cyclic pyranopterin monophosphate (cPMP, precursor Z) into MPT [27]. MPT constitutes the molybdenum cofactor (Moco), which is essential for the activity of several enzymes, including xanthine dehydrogenase, aldehyde oxidase, sulfite oxidase and mARC [9, 10]. Impaired Moco biosynthesis reduces xanthine oxidoreductase activity, leading to disrupted purine catabolism with decreased uric acid and accumulation of hypoxanthine and xanthine. In addition, deficiency of sulfite oxidase activity results in impaired sulfur detoxification and accumulation of sulfite-derived metabolites such as S-sulfocysteine (SSC), which contribute to neuronal and glial injury and manifest clinically as seizures, developmental delay and progressive white-matter loss. In this study, we described two children with mild MoCD-B carrying biallelic *MOCS2* variants—one with a homozygous nonsense variant c.16 C > T (p.Gln6Ter) and the other with compound heterozygous variants c.16 C > T and the novel missense change c.257G > T (p.Ser86Ile). In addition to the novel c.257G > T mutation, our cases provide the first evidence that SARS-CoV-2 infection may trigger an acute encephalopathic episode in mild MoCD-B and were accompanied by elevated CSF IL-8.

Mild or late-onset forms of MoCD typically present in infancy but follow a milder course than the classic neonatal phenotype. A characteristic clinical feature is infection-associated neurological decompensation, presenting with episodic altered consciousness, dystonia and choreoathetosis. These features may partially improve after recover of the inciting infection, or progress gradually or unpredictably over the lifetime. Neuroimaging can be normal or show T2-weighted hyperintensities or cystic changes in the globus pallidus, thinning of the corpus callosum and cerebellar atrophy [27], while laboratory findings such as hypouricemia and accumulation of hypoxanthine, xanthine or SSC strongly support the diagnosis. Genetic confirmation is established by identifying biallelic pathogenic or likely pathogenic variants in *MOCS1*, *MOCS2*, *MOCS3*, or *GEPH*. Within this spectrum, mild MoCD-B caused by *MOCS2* variants shows a broadly similar pattern. We reviewed previously reported 11 patients and found that infection-triggered acute neurological decompensation, seizures, motor dysfunction, facial dysmorphism, and abnormal MRI findings are common, with lesions of the globus pallidus being the most frequent radiological feature. In contrast to mild MoCD across all genetic subtypes, neuroimaging involvement except the globus pallidus in mild MoCD-B is less consistent: cerebral peduncle involvement, cortical dysplasia, and cerebral atrophy are variably reported, whereas dentate nucleus lesions, diffuse white-matter involvement, and corpus callosum thinning are only occasionally observed.

Build on the background of infection-triggered neurological decompensation mild MoCD-B, our two patients further expand the spectrum of precipitating infections by suggesting that SARS-CoV-2 can serve as a trigger for acute encephalopathic episodes. Both children developed acute encephalopathy shortly after COVID-19, followed by dystonia, seizures, motor regression. However, the distribution of brain lesions—predominant T2 hyperintensities in the globus pallidus and cerebral peduncles—did not match the typical neuroimaging patterns of COVID-19–associated encephalopathies such as acute necrotizing encephalopathy, acute disseminated encephalomyelitis or reversible splenial lesion syndrome [28]. Although the initial course of immunotherapy led to partial clinical improvement, the persistence and progression of neurological deficits, together with a history of developmental delay, ultimately pointed to an underlying metabolic disorder rather than isolated viral encephalitis.

Both patients showed elevated IL-8 and other pro-inflammatory cytokines in CSF during the acute phase. Although IL-8 as a neutrophil-recruiting chemokine, has been associated with encephalopathy severity and prognosis in patients with ANE [29], other explanations including infection-driven cytokine release and immune activation independent of MoCD-B also should be considered, such as sterile neuroinflammation related to hypoxia/ischemia or toxic-metabolic stress. It is important to note that elevated IL-8 may reflect a non-specific response to acute infection rather than a disease-specific pathogenic mediator. Large cohorts and longitudinal sampling are needed to determine whether IL-8 is consistently elevated in mild MoCD-B or merely reflects intercurrent infection.

To explore the pathogenicity of the two variants identified in our patients, we reviewed previously reported *MOCS2* variants. To date, 15 variants have been reported, including seven missense, six nonsense, and two deletion variants. In our study, both patients with mild MoCD-B carried the nonsense variant c.16 C > T (p.Gln6Ter) in *MOCS2A*—in homozygous form in one patient and in compound heterozygous form with the novel missense change c.257G > T (p.Ser86Ile) in the other. Previous reports have identified c.16 C > T in four additional patients with mild MoCD-B, all of Chinese origin [4, 13, 14]. Although homozygous c.16 C > T is generally associated with a mild phenotype, one Chinese family from the Taiwan region showed intrafamilial variability, with one sibling exhibiting a mild course and the other manifesting a more typical neonatal-onset phenotype but with relatively prolonged survival [14], suggesting that genetic background and/or additional modifier factors may modulate disease severity. The c.257G > T variant is absent from population databases. In the affected individual, it was detected in trans with another pathogenic *MOCS2* allele (c.16 C > T) consistent with the recessive inheritance pattern. Multiple in silico prediction tools suggested a deleterious effect on the protein. Clinically, the patient’s phenotype and biochemical profile were highly specific for MoCD-B. Accordingly, c.257G > T was classified as likely pathogenic (PM2 + PM3 + PP3 + PP4) under ACMG criteria.

To better understand why our patients followed a relatively mild course without biochemical evidence of MPT synase, we next focused on the possible structural and functional changes of missense *MOCS2* variants. Among the seven missense variants in *MOCS2A*, p.Ser86Ile, p.Gly88Ala, and p.Val7Phe have been reported to be associated with mild phenotypes; in our structural modeling, we predicted that these variants caused only limited local conformational changes, either at the MOCS2A–MOCS2B interface (p.Ser86Ile, p.Gly88Ala) or in the N-terminal region (p.Val7Phe). As a novel variant identified in our study, the p.Ser86Ile variant locates at the MOCS2A–MOCS2B interface, which is predicted in our structural modeling to cause limited local distortion around C ternimal. The p.Gly88Ala variant is particularly informative, as it is located at the terminal residue of the Gly–Gly motif. Although bacterial mutagenesis at the glycine residue equivalent to human Gly88 suggests almost complete loss of detectable activity when mutated [30], the association of p.Gly88Ala with a mild phenotype in humans may indicate residual in vivo activity and highlight potential species-dependent effects. Finally, for p.Val7Phe, prior functional studies demonstrated impaired complex formation by chromatography and reduced in vitro MPT synthase activity (~ 10% of wild-type at 120 min), with accumulation of a half-sulfurated intermediate [31], supporting a hypomorphic effect. By contrast, four other missense variants (p.Ser15Arg, p.Leu73Pro, p.Gly76Arg and p.Ile82Phe), reported in patients with severe MoCD-B, are predicted to result in relatively marked local structural alterations, including reduced hydrogen bonding and/or changes in side-chain charge or volume that distort local packing. Among them, the p.Ser15Arg mutant protein could not be successfully expressed or purified in E. coli, indicating a profound destabilizing effect on protein folding and stability [31]. Thus, we predicted these pronounced structural changes, impairing insertion of the MOCS2A C-terminal Gly-Gly motif into the MOCS2B pocket, thereby disrupting its sulfur-transfer function. Why do p.Ser86Ile and p.Gly88Ala, despite being proximal to or within the critical C-terminal Gly–Gly sulfur-donor motif, associate with a mild phenotype? Although p.Ser86Ile and p.Gly88Ala mapped to the MOCS2A–MOCS2B interface near or within the C-terminal Gly–Gly motif, both changes were predicted to be relatively non-disruptive at the level of tail insertion. p.Gly88Ala substitution is a conservative change with minimal steric burden. We therefore speculated that the C-terminal tail could still access the MOCS2B pocket and support partial sulfur transfer, resulting in a hypomorphic allele with residual activity that is sufficient to produce a mild phenotype, whereas severe variants are predicted to prevent accurate insertion of the thiocarboxylated terminus into the MOCS2B catalytic pocket, thereby interrupting sulfur transfer and ultimately abolishing or severely impairing MPT synthase activity. Nevertheless, the effects of missense variants on MPT synthase assembly and catalytic function need further experimental validation.

From a therapeutic perspective, no specific treatment can be used for patients with MoCD-B, AAV related therapy has been explored in animal models [32]. In this study, both patients received immunotherapy with IVIG and/or methylprednisolone and showed clinical improvement. Our observations do not support routine immunotherapy but suggest that, in certain cases, treatment decisions should align with established guidelines for COVID-19–related neurological complications [33]. In previously reported mild MoCD-B, the recovery course is variable: patients typically need several months to reach a relatively stable condition, recovery may take up to two years, and many do not return to their pre-infection baseline but rather progress slowly to a plateau. In our patient, intravenous immunoglobulin was given primarily to modulate the immune response and elevated cytokine triggered by SARS-CoV-2 infection, aiming to promote recovery from the acute encephalopathic state rather than to treat the underlying MoCD-B itself. To date, besides our patients, only one patient has been reported who received a single IVIG course (2 g/kg) and seemed had worse muscle tone recovery than our Patient 1. However, additional cases will be required to determine whether immunotherapy can consistently accelerate recovery in this setting. The infection-related neurological deterioration in our two patients before and after Covid-19 infection, underscores the importance of preventing SARS-CoV-2 exposure. This study is limited by the small sample size, lack of longitudinal data, and absence of functional validation for the identified variants. Larger, multicenter studies with long-term follow-up and functional assays are needed to confirm our findings and further investigate the disease mechanisms and treatment options.

## Conclusion

This study expands the genetic spectrum of MoCD-B by identifying for the first time a novel *MOCS2* variant (c.257G > T, p.Ser86Ile). In infants, acute neurological deterioration after infection accompanied by persistent hypouricemia and bilateral globus pallidus lesions should prompt consideration of mild MoCD-B. Our observations do not provide sufficient evidence to support routine immunotherapy for MoCD-B patients after SARS-CoV-2 infection; treatment management should be guided by established recommendations for COVID-19–associated neurological complications.

## Supplementary Information

Supplementary Material 1.

Supplementary Material 2.

Supplementary Material 3.

Supplementary Material 4.

Supplementary Material 5.

Supplementary Material 6.

Supplementary Material 7.

## Data Availability

The datasets of variants in patients generated and/or analyzed during the current study are available in the GenBank repository. [Accession number PX130405 and PX130406.[https://www.ncbi.nlm.nih.gov/nuccore/PX130405; https://www.ncbi.nlm.nih.gov/nuccore/PX130406].

## Abbreviations

SARS-CoV-2: Severe acute respiratory syndrome coronavirus 2
ANE: Acute necrotizing encephalopathy
RANBP2: Ran Binding Protein 2
MoCD: Molybdenum cofactor deficiency
MOCS2: Molybdenum cofactor synthesis gene 2
MRI: Magnetic resonance imaging
MOCS1: Molybdenum cofactor synthesis gene 1
Moco: Molybdenum cofactor
MOCS2A: Molybdenum cofactor synthesis gene 2 A
MOCS2B: Molybdenum cofactor synthesis gene 2B
MPT: Molybdopterin
cPMP: Cyclic pyranopterin monophosphate
LSS: Leigh Syndrome Spectrum
SSC: S-Sulfocysteine
VUS: Uncertain significance
CSF: Cerebrospinal fluid
EEG: Electroencephalogram
DWI: Diffusion weighted imaging
ADC: Apparent diffusion coefficient
IVIG: Immunoglobulin
MRA: Magnetic resonance angiography
BAEP: Brainstem auditory evoked potential
IL-6: Interleukin-6
IL-8: Interleukin-8
MP: Methylprednisolone
GC-MS: Gas chromatography-mass spectrometry
WES: Whole-exome sequencing
DD: Developmental delay
ISOD: Isolated sulfite oxidase deficiency
