# Supplementary material for: Two mild phenotype molybdenum cofactor deficiency patients with novel MOCS2 mutation and immunological treatment after COVID-19 infection
Source: BMC Neurol. 2026 Feb 23;26:266. doi: 10.1186/s12883-026-04697-9 (PMC13104280; doi:10.1186/s12883-026-04697-9)
Supplement: Supplementary file 1 — Supplementary Material 1. [file 12883_2026_4697_MOESM1_ESM.docx]

**Supplementary Information**

**Table S1** General clinical data of 13 mild patients with MoCD typeB.

Among them, 10 were female and 3 were male. The onset age ranged from neonatal to 72 months, with a median age of 7.5 months. 92.3% (12/13) patients presented before 2 years old. Only one patient developed lens dislocation and cranial imaging change in school age, without significant clinical effects until early adulthood. The initial symptoms are heterogeneous, including developmental delay (DD, 3/13, 23%), behavioral abnormalities (2/13, 15.4%), seizures (3/13, 23%), dystonia (1/13, 7.7%), hemiparesis (1/13, 7.7%), motor regression (2/13, 15.4%) and lens dislocation (1/13, 7.7%). Neurological signs including extrapyramidal sign (11/12, 91.6%), epilepsy (6/13, 46.1%), feed difficulty (8/13, 61.5%) and pyramidal sign (7/11, 63.6%), and abnormal behavior (6/12, 50%) were prominent. With the progression of disease, DD (9/13, 69.2%), motor delay (9/13, 69.2%), and language delay were common, yet some patients retained partial function, 50% (5/10) spoke simple sentences; 46.1% (6/13) walked independently. Retrogression occurred in 81% patients (9/11) after disease onset. Five patients (6/13, 46.1%) presented with facial dysmorphism, and individual cases showed either macrocephaly or microcephaly. Infection-triggered onset (9/12, 75%), Lens dislocation (3/8, 37.5%), and hypermetropia (2/8, 25%) were observed.

EEG abnormalities occurred in 81.8% (9/11), featuring slow waves in 3 cases; 2 showed normal EEGs. Serum uric acid levels were reduced in 81.8% (9/11) of patients, Urinary uric acid excretion was decreased in all tested cases (2/2). Serum cysteine and homocysteine levels decreased in in 50% (2/4) of patients each. All tested patients showed elevated s-sulfocysteine levels in both plasma (2/2) and urine (4/4), and urine sulfite strips were positive in all cases (5/5). Urinary urate levels were decreased in 33.3% (1/3) of patients. Serum xanthine and hypoxanthine levels were elevated in all tested patients (3/3 and 2/2, respectively). Similarly, urinary xanthine levels were elevated in all cases (6/6), while hypoxanthinuria was present in 80% (4/5) of patients.

All patients exhibited abnormalities in MRI, predominantly T2WI hyperintensity in the GP (10/13,76.9%), followed by CP (4/13,30.7%), cortical dysplasia (3/13, 23%), dentate nucleus (2/13, 15.4%), cystic cavitation in the GP (2/13, 15.4%), white matter (2/13, 15.4%), corpus callosum thinning, caudate and lentiform nuclei (1/13, 7.7%). Atrophy was noted in 3 patients (3/13, 23%).

**Figure S1.** Sanger sequencing of two missense variants in *MOCS2* in this study.

a-c variant of *MOCS2A* in Pt1’s family, a. sequencing of Pt1, b. sequencing of Pt1’s mother; c. sequencing of Pt1’s father. d-i. Variants of *MOCS2A* in Pt2’s family. d-e, sequencing of Pt2, f-g. sequencing of Pt2’s mother; h-i. sequencing of Pt2’s father.

**Figure S2.**

Figure S2. MRI of Pt1 and Pt2.
a-e MRI of Pt1 at 6 months 7 days; a-c Bilateral diffuse abnormal signals in globus pallidus in T2WI, DWI and ADC (red arrows), and extracerebral space (green arrow heads); d-e No abnormal signals in bilateral cerebral peduncles was detected in T2WI and DWI (red arrows);

f-j MRI of Pt1 at 6 months 15 days; f-h, Narrowed bilateral diffuse abnormal signals in globus pallidus in T2WI, DWI and ADC (red arrows); i-j, Enlarged abnormal signals in bilateral cerebral peduncles were detected in axial T2WI and DWI (red arrows);

k-o MRI of Pt2 at 7 months 9 days, k-m, Bilateral diffuse abnormal signals in globus pallidus in T2WI, DWI and ADC (red arrows); n-o, Bilateral diffuse abnormal signals in cerebral peduncles were detected in T2WI and DWI (red arrows);

p-t MRI of Pt2 at 7 months 25 days; p-r, Enlarged bilateral diffuse abnormal signals in globus pallidus in axial T2WI, DWI, ADC and T1WI (red arrows); s-t, Enlarged diffuse abnormal signals in cerebral peduncles were detected in T2WI and DWI (red arrows); p, enlarged extracerebral space (yellow arrow heads).

**Figure S3.** Structural consequences of missense variants in MOCS2 protein.

(A) In the wild-type (WT) model, Val7 forms two hydrogen bonds with Ile26; after substitution (Val7Phe), the number of hydrogen bonds remains unchanged.

(B) In WT, Leu73 forms one hydrogen bond with Gln30, which is lost in the Leu73Pro mutant.

(C) In WT, Gly76 forms one hydrogen bond with Gln6; after substitution (Gly76Arg), the number of hydrogen bonds is unchanged, although the residue changes from neutral glycine to a positively charged arginine.

(D) In WT, Ile82 forms two hydrogen bonds with Ile56; the number of hydrogen bonds remains unchanged in the Ile82Phe mutant.

(E) In WT, Ser15 forms a total of seven hydrogen bonds with Val81, Phe12, Thr19, and Ala16. After substitution (Ser15Arg), the hydrogen-bonding pattern is redistributed: the interaction with Ala16 is lost, while bonds are retained/formed with Phe12 (1), Val81 (2), and Thr19 (2).
