# Supplementary figures and images for: Two mild phenotype molybdenum cofactor deficiency patients with novel MOCS2 mutation and immunological treatment after COVID-19 infection
Source: BMC Neurol. 2026 Feb 23;26:266. doi: 10.1186/s12883-026-04697-9 (PMC13104280; doi:10.1186/s12883-026-04697-9)

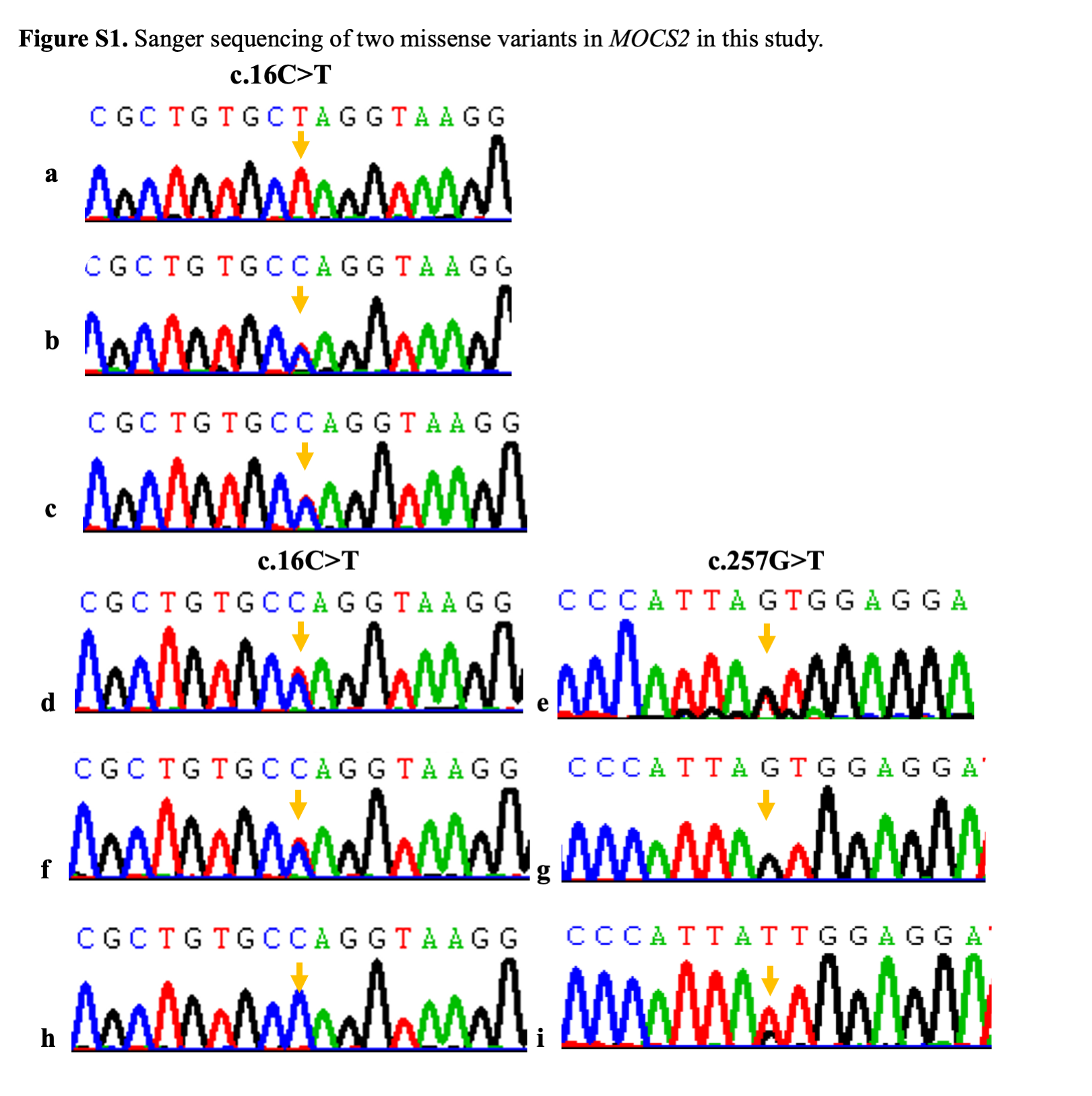

Supplement: Supplementary file 2 — Supplementary Material 2. [file 12883_2026_4697_MOESM2_ESM.png]

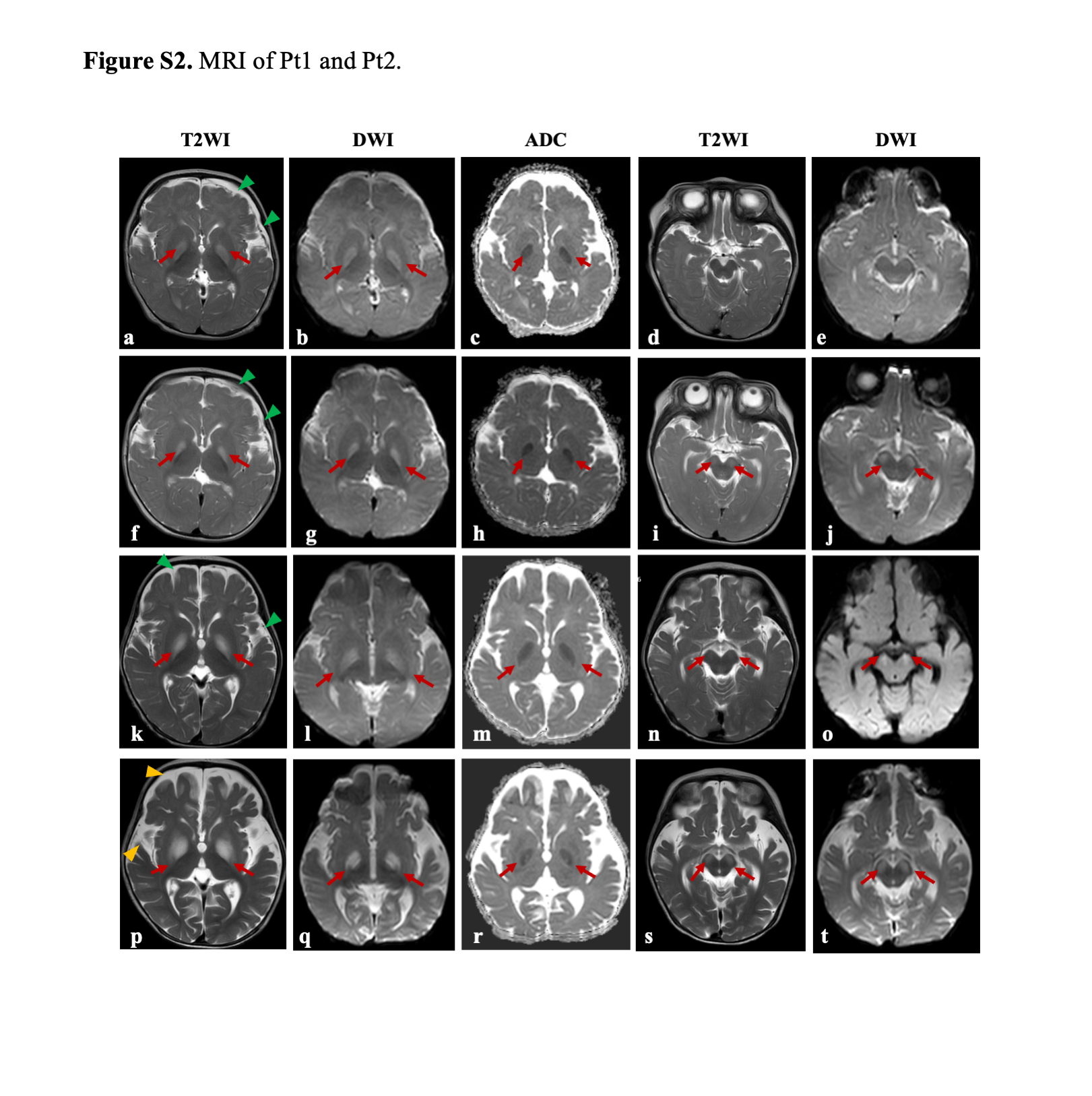

Supplement: Supplementary file 3 — Supplementary Material 3. [file 12883_2026_4697_MOESM3_ESM.png]

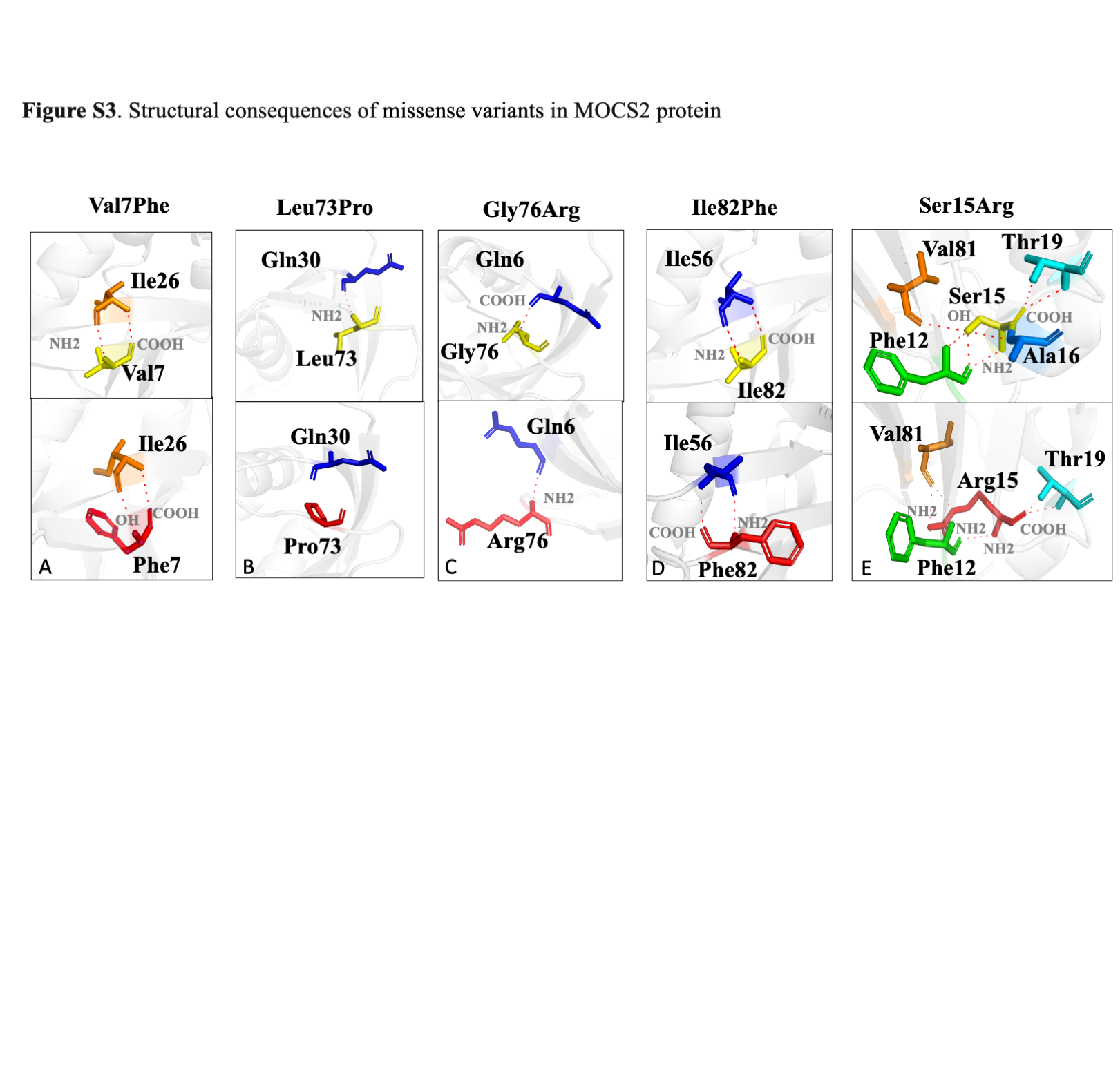

Supplement: Supplementary file 4 — Supplementary Material 4. [file 12883_2026_4697_MOESM4_ESM.png]
