# Supplementary material for: Two mild phenotype molybdenum cofactor deficiency patients with novel MOCS2 mutation and immunological treatment after COVID-19 infection
Source: BMC Neurol. 2026 Feb 23;26:266. doi: 10.1186/s12883-026-04697-9 (PMC13104280; doi:10.1186/s12883-026-04697-9)
Supplement: Supplementary file 5 — Supplementary Material 5. [file 12883_2026_4697_MOESM5_ESM.docx]

Table S1 General clinical data of 13 mild patients with MoCD typeB.

| article | This article | | | Hughes EF et al. (1998) | Alkufri F et al.  (2013) | Johnson JL et al. (2001) | Zaki MS et al. (2016) | Megahed H et al.  (2016) | Duman et al.  (2018) | Scelsa B et al.  (2019) | Tian XJ et al.  (2021) | Lee HF et al.  (2021) | Yan W et al.  (2022) | Kinsinger M et al. |
| --- | --- | --- | --- | --- | --- | --- | --- | --- | --- | --- | --- | --- | --- | --- |
|  | Patient 1 | Patient 2 | |  |  |  |  |  |  |  |  |  |  | (2023) |
| Gender | female | | female | female | female | female | female | female | female | female | male | male | male | female |
| Race | Chinese | | Chinese | white | white | Chinese | Egyptian | Egyptian | Turkish | Chinese | Chinese | Chinese (Taiwan) | Chinese | Pakistani |
| Onset age (months) | 5 | | 6 | 12 | 72 | 15 | 6 | 0 | 5 | 16 | 7.5 | 17 | 4 | 12 |
| Onset symptom | DD | | DD | abnormal behavior | bilateral lens dislocation | dystonia | seizure | DD | seizure | left hemiparesis | abnormal behavior | motor retrogression | seizure |  |
| Infectious disease before onset | + | | + | + | NA | + | + | NA | NA | + | + | + | NA | + |
| Development delay | + | | + | + | - | + | + | + | + | + | - | - | + | - |
| Retrogression after intercurrent illness | + | | + | + | + | + | + | - | NA | - | + | + | NA | + |
| Seizure | - | | - | + | - | - | + | + | + | + | - | - | + | - |
| Feeding difficulties | + | | + | + | + | - | + | - | - | - | + | - | + | + |
| Behavioral disorders | + | | + | + | + | - | - | + | - | - | + | - | NA | - |
| Facial deformity | + | | + | - | - | macrocephaly | +microcephaly | + | + | - | - | - | +microcephaly | - |
| Language development | / | | / | Normal, regression | Normal, regression | delay (expressive language) | delay | delay (expressive language) | delay (5-6 words) | delay (expressive language) | / | delay | delay | normal, regression |
| Motor development | delay, can't sit | | delay, can't sit | delay, can't walk | walk, regression after 23 years of age | delay, regression, walk | delay, can't walk | delay, walk (wide gait) | delay, sit | delay, walk | normal, regression-can't sit | normal, walk, but regression | delay, can't sit | normal, regression, walk |
| Extrapyramidal signs | + | | + | + | + | + | + | - | + | + | + | + | NA | + |
| Pyramidal signs | + | | - | - | - | - | + | + |  | + | + | + | + | NA |
| Ophthalmological examination | NA | | NA | - | dislocation of the lens | hypermetropia | - | NA | - | hypermetropia | NA | NA | Ectopia lentis, Spherophakia, Retinal detachment,Hyphema, Secondary glaucoma Cyclodialysis | Strabismus |
| EEG | slow wave | | slow wave | abnormal | NA | normal | Focal | Left fronto-temporal epileptogenic dysfunction | dislocation of the lens | sharp delta activity by sleep over the right parieto‐temporal area | normal | NA | abnormal | 6 Hz PDR bursts of slowing |
| Cytokine | + | | + | - | negative | / | / | / | asynchronous multifocal slow-wave activity | / | / | / | NP | NP |
| MRI | GP; CP; cortical dysplasia | | GP; CP; cortical dysplasia; atrophy | caudate, lentiform nuclei; CP; DN | GP | GP; cortical dysplasia | GP; thinned CC; white matter volume loss; atrophy | cerebellar vermis atrophy | GP | GP; cystic cavitation in GP; DN | GP; CP | GP, a cystic cavitation in GP; cerebellar hemisphere | Symmetric subcortical cystic leukomalacia; enlarged cisterna magna | GP |
| Plasma uric acid level | ↓ | | ↓ | ↓ | ↓ | normal | normal | NA | ↓ | ↓ | ↓ | ↓ | NA | ↓ |
| Plasma xanthine/hypoxanthine | NP | | NP | ↑/↑ | ↑/↑ | NP | NP | ↑/NA | NA | NP | NP | NA | NA | NA |
| Serum amino acid | (+ ) | | -(not full spectrum) | normal | NA | / | NP | NP | NA | +Cystine↓ | methionine, leucine, valine, arginine↑ | NP | NA | (+) |
| Plasma S‐sulfocysteine | NP | | NP | NA | NA | ↑ | NP | ↑ | NA | NA | NP | NP | NA |  |
| Urine sulfocysteine | NP | | NP | NP | NP | NP | ↑ | NP | NA | ↑ | NP | ↑ | NA |  |
| Urine sulfite strip test | NP | | NP | + | + | + | NP | NA | NA | + | NP | + | NA |  |
| Urine uric acid | NP | | NP | ↓ | ↓ | NP | NP | NP | NA | NP | NP | NP | NA | NP |
| Urine xanthine/hypoxanthine | NP | | NP | ↑/↑ | ↑/↑ | ↑/normal | ↑/NP | NP | NA | NP | ↑/↑ | NP | NA | ↑/↑ |
| Urine GCMS | - | | - | - | NA | NP | NP | NP | NA | Cystine↓ | phenyllactic acid, 4-Hydroxyphenyllactic acid↓ | NP | NP | NP |
| Treatment | two course IVIG; MP; nitrazepam，coenzyme Q10, energy mixture, | | two course IVIG; MP; coenzyme Q10, diazepam, midazolam, Levetiracetam | carbamazepine | a short trial of oral prednisolone; levodopa | no treatment | no respond to ASM | sodium valproate, Levitiracetam, omega 3 supplements, and intra-muscular B complex. | Levitiracetam | acetylsalicylic acid | IVIG; MP; L-carnitine, coenzyme Q10, B-Complex Vitamin Tablet, benzhexol, levodopa, clonazepam | - | ASM | NA |
| Outcome at last visit age | severe (1.25) | | severe, died of respiratory problem (0.67) | severe (3) | severe (23) | mild (4.5) | severe (died at 5.5 years old) | mild (6) | mild | mild (6) | severe (0.5) | severe (3) | severe (6) | severe (3) |
| Nucleotide variants | c.16C>T/ c.16C>T | | c.16C>T/c.257G>T | c.564G>C; c.726-727 delAA | c.564G>C; c.726-727 delAA | c.16C>T/c.19G>T | c.3G > A/ c.3G > A | c.3G > A/ c.3G > A | c.3G > A/ c.3G > A | c.19G>T/ c.19G>T | c.19G>T/c.19G>T | c.16C>T/ c.16C>T | c.16C>T/ c.16C>T | c.263G > C/ c.263G > C |

CC, corpus callosum; DD, developmental delay; GP, globus pallidus; CP, cerebral peduncle; MP, methylprednisolone; IVIG, intravenous immunoglobulin; ASM, antiseizure medi cation; NA, not available; NP, not performed.
