# Supplementary material for: Two mild phenotype molybdenum cofactor deficiency patients with novel MOCS2 mutation and immunological treatment after COVID-19 infection
Source: BMC Neurol. 2026 Feb 23;26:266. doi: 10.1186/s12883-026-04697-9 (PMC13104280; doi:10.1186/s12883-026-04697-9)
Supplement: Supplementary file 6 — Supplementary Material 6. [file 12883_2026_4697_MOESM6_ESM.docx]

Table S2. Reference Ranges of cytokines.

| Cytokines | Reference Range (pg/ml) |
| --- | --- |
| IFN-γ | 0-4.43 |
| IL-1β | 0-3.40 |
| IL-4 | 0-4.19 |
| IL-5 | 0-4.15 |
| IL-6 | 0-11.09 |
| IL-10 | 0-4.50 |
| IL-17A | 0-4.74 |
| IL-22 | 0-3.64 |
| TNF-α | 0-4.50 |
| IL-8 | 0-15.71 |
| IL-2 | 0-6.64 |
| TNF-β | 0-2.54 |
| IL-12p70 | 0-10.18 |
| IL-17F | 0-4.66 |
