# Supplementary material for: Two mild phenotype molybdenum cofactor deficiency patients with novel MOCS2 mutation and immunological treatment after COVID-19 infection
Source: BMC Neurol. 2026 Feb 23;26:266. doi: 10.1186/s12883-026-04697-9 (PMC13104280; doi:10.1186/s12883-026-04697-9)
Supplement: Supplementary file 7 — Supplementary Material 7. [file 12883_2026_4697_MOESM7_ESM.docx]

Table S3 Neurological function recording around SARS-CoV-2–associated exacerbation in mild MoCD-B.

|  | Time point | Consciousness | Swallowing function | Gross motor function | Fine motor function | Muscle strength | Muscle tone |
| --- | --- | --- | --- | --- | --- | --- | --- |
| Patient 1 | Before infection (6m) | *** | *** | ** | *** | *** | *** |
|  | Discharge from hospital (7m) | *** | ** | * | * | ** | ** |
|  | 3 months after discharge (11m) | *** | ** | *- | * | ** | * |
| Patient 2 | Before infection (7m) | *** | *** | ** | *** | *** | ** |
|  | Discharge from hospital (9m) | *** | * | * | * | * | * |

*** Neurological functions were assessed semi-quantitatively using an ordinal scale. “*******” indicates age-appropriate or near-normal function;

“**” indicates mild impairment; “*” indicates moderate to severe impairment. Assessments were based on functional observations at each time point.
